# High Cardiorespiratory Fitness Is Negatively Associated with Daily Cortisol Output in Healthy Aging Men

**DOI:** 10.1371/journal.pone.0141970

**Published:** 2015-11-03

**Authors:** Francesco Lucertini, Elisa Ponzio, Michael Di Palma, Claudia Galati, Ario Federici, Pamela Barbadoro, Marcello M. D’Errico, Emilia Prospero, Patrizia Ambrogini, Riccardo Cuppini, Davide Lattanzi, Andrea Minelli

**Affiliations:** 1 Department of Biomolecular Sciences, Division of Exercise and Health Sciences, University of Urbino Carlo Bo, Urbino, Italy; 2 Department of Biomedical Sciences and Public Health, Università Politecnica delle Marche, Ancona, Italy; 3 Department of Earth, Life and Environmental Sciences (DiSTeVA), University of Urbino Carlo Bo, Urbino, Italy; University of Marburg, GERMANY

## Abstract

Physical fitness has salutary psychological and physical effects in older adults by promoting neuroplasticity and adaptation to stress. In aging, however, the effects of fitness on the hypothalamic-pituitary-adrenal (HPA) axis are mixed. We investigated the association between cardiorespiratory fitness and HPA activity in healthy elderly men (*n* = 22, mean age 68 y; smokers, obese subjects, those taking drugs or reporting recent stressful events were excluded), by measuring in saliva: i) daily pattern of cortisol secretion (6 samples: 30’ post-awakening, and at 12.00, 15.00, 18.00, 21.00, 24.00 h); and ii) the cortisol response to a mental challenge. Cardiorespiratory fitness (VO_2max_) was estimated using the Rockport Walking Test and the participants were assigned to high-fit (HF, ≥60°, *n* = 10) and low-fit (LF, ≤35°, *n* = 12) groups according to age-specific percentiles of VO_2max_ distribution in the general population. At all daytimes, basal cortisol levels were lower in the HF than the LF group, most notably in the evening and midnight samples, with a significant main effect of physical fitness for cortisol levels overall; the area-under-the-curve for total daily cortisol output was significantly smaller in the HF group. Among the subjects who responded to mental stress (baseline-to-peak increment >1.5 nmol/L; *n* = 13, 5 LF, 8 HF), the amplitude of cortisol response and the steepness of recovery decline displayed an increasing trend in the HF subjects, although between-group differences failed to reach the threshold for significance. In conclusion, cardiorespiratory fitness in healthy aging men is negatively correlated with daily cortisol output and contributes to buffering the HPA dysregulation that occurs with advancing age, thus possibly playing a beneficial role in contrasting age-related cognitive and physical decline.

## Introduction

Aging is a complex, multifactorial process which shows great individual variability. The trajectory of aging could be related to a variety of factors, including individual differences in the hypothalamic-pituitary-adrenal (HPA) axis activity [1, 2]. Cortisol, the end-product of the HPA axis, acts as a key regulator of inflammation and metabolic activity, as well as a primary agent of the neuroendocrine stress response. Across the day, cortisol secretion levels follows a typical circadian rhythm, sharply increasing within 1 hour after waking and steadily declining thereafter, reaching a nadir in the late evening hours [3]. Major changes in this diurnal pattern have consistently been observed with aging. Age-related increase of daytime plasma cortisol levels has been documented using multiple blood sampling [4, 5]. These findings have been extended by using salivary sampling method as a non-invasive tool for free cortisol assessment, allowing for frequently repeated measures in more naturalistic settings. In community-dwelling older adults, diurnal cortisol levels and total cortisol output (as the area under the curve, AUC) have been reported as increasing with advancing age [6]. Studies using multiple daily assessments over a period of several days have confirmed these findings: in a group of 185 healthy adults of both sexes, age was found to be associated with an increased daily cortisol AUC, an attenuated wake-evening slope, and a greater cortisol awakening response [7]. Evidence from a sample of 1963 men and women also showed that older age and male gender are independently associated with higher cortisol peak, nadir and AUC [8]. Interestingly, stress-related psychosocial and affective dimensions seem to play a role in moderating age-related changes in HPA activity: in a large sample spanning 50 years of adulthood, the increase in cortisol output across the day was shown to correlate with higher levels of average negative affect [9]; in older adults, notably those reporting high levels of perceived stress, decline in self-esteem has been shown to predict an elevated diurnal cortisol output [10]; a 4-year longitudinal study involving 157 community-dwelling aged persons showed that levels and increases of sleep duration buffered the long-term elevation of diurnal cortisol secretion [11]. Age-related effects on cortisol secretion have mainly been ascribed to the impairment of feedback inhibition of HPA activity due to neuronal loss within specific brain areas, such as prefrontal cortex and hippocampus, which exert an inhibitory action on stress-sensitive neurons in the amygdala and hypothalamus [12–14]. Importantly, the dysregulation of the HPA axis is regarded as a psychobiological mechanism underlying age-related psychophysical decline [15]. Frequent HPA activation and increased cortisol secretion are proposed as being etiological in the development of several chronic conditions that become manifest in the older population, including cardiovascular disease [16], cognitive decline [17–19], depression [20, 21], and frailty [22, 23].

Another important factor in aging is physical fitness. In older individuals, an active lifestyle is associated with a higher quality of life [24], and has salutary psychological and physical effects [25]. In aging, a physically active life and/or a high level of cardiorespiratory fitness yield positive effects on brain plasticity and regional grey matter volume, most notably in prefrontal and hippocampal areas [26], thus providing a biological basis for their beneficial influence on cognitive and affective domains, and promoting better control of the HPA axis and greater resilience to stress [13]. However, studies that evaluated the effects of aerobic fitness on HPA axis activity in an aging population have produced conflicting results: no significant differences in serum or salivary cortisol concentrations were observed in life-time exercisers compared to sedentary elderly men, despite remarkable between-group differences in maximal oxygen consumption (VO_2max_) (but cortisol was only measured once) [27, 28]. In contrast, habitual physical activity was shown to buffer against the negative effects of stress in older men and women [29] by opposing the stress-associated increase in the ratio between salivary levels of cortisol and those of dehydroepiandrosterone (DHEA), a hormone of the HPA axis acting as an endogenous glucocorticoid antagonist, with wide-ranging positive effects on immunomodulation and well-being [30] (although VO_2max_ was not assessed in this study [29]). Results from intervention studies are also inconclusive: a 6-month aerobic exercise program that increased performance and VO_2max_ in older adults of both sexes led to a reduction of plasma cortisol levels only in women [31]. Moreover, a 6-week period aerobic training in sedentary aging men, although increasing VO_2max_, failed to affect salivary cortisol output [32]. In both studies, however, only a single morning measure of cortisol was collected. Acute HPA reactivity to stressful challenges has also been reported to be influenced by physical fitness, with high cardiorespiratory fitness being associated with a blunted cortisol response to psychological stress among older women [33]. Recent studies, however, have failed to replicate such a finding in younger adults [34, 35].

In the current study, the issue of the association between physical fitness and HPA axis activity was reinvestigated in a group of healthy elderly men to assess whether cardiorespiratory fitness levels, indexed by estimating VO_2max_, are correlated with differences in HPA axis activity under both basal and stimulated conditions. This was achieved by measuring in saliva samples: i) daily pattern of basal cortisol secretion; and ii) the cortisol response to a psycho-cognitive challenge.

## Materials and Methods

### Participants

**Fig 1:**
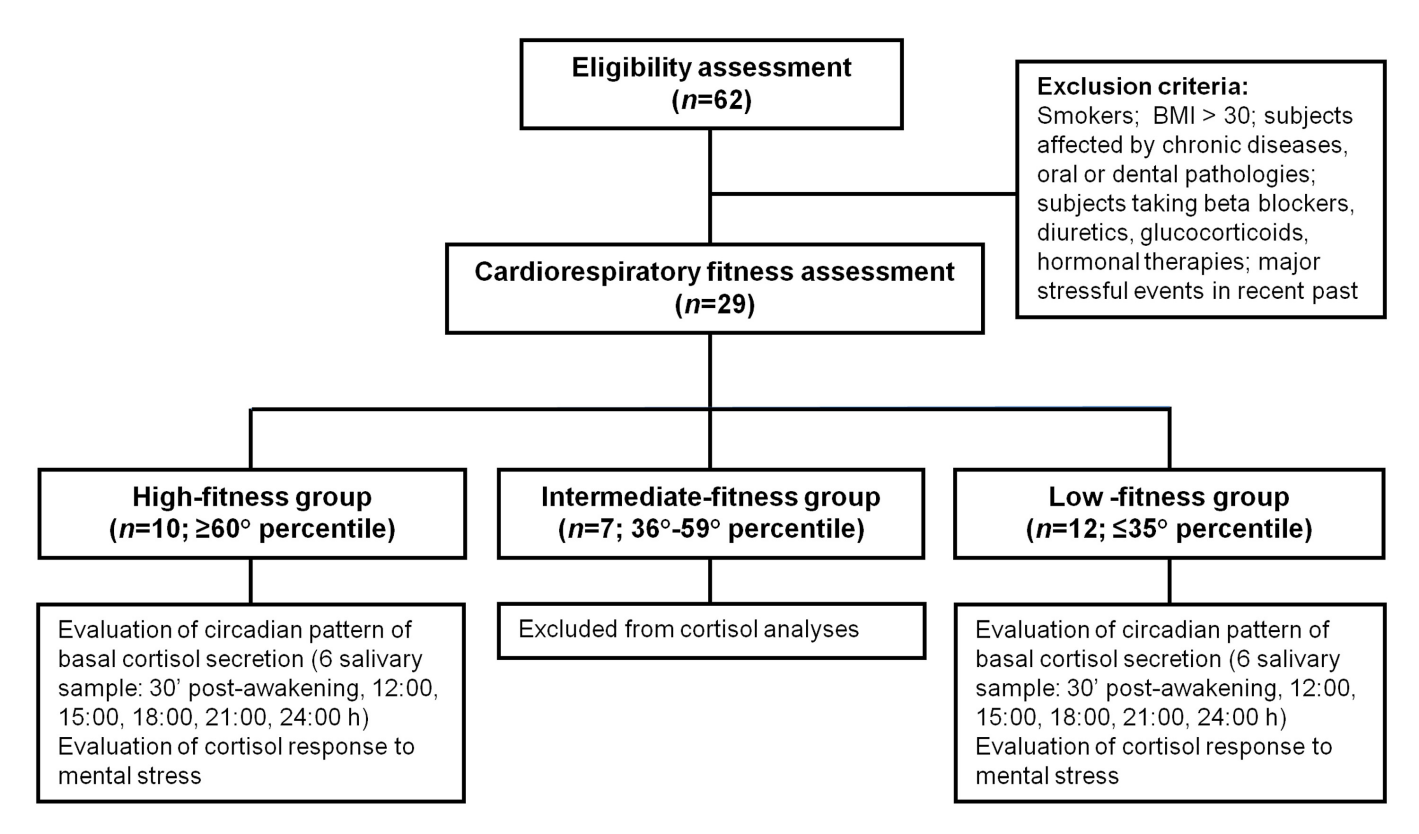
Flowchart of the study participants. Age-specific percentiles for cardiorespiratory fitness were retrieved from [36].

Healthy elderly men were recruited from various senior citizen centers, all located in the area of Urbino (Marche Region, Italy). The initiative was advertised by posting circulars. Subjects willing to participate were given thorough explanations describing the design and aims of the study, and the experimental methods and procedures. Phone numbers were provided for prospective participants who wanted additional information. Smokers, the obese (BMI>30), subjects affected by chronic diseases or oral/dental pathologies, people taking beta blockers, diuretics, or glucocorticoids, or those reporting bereavements or major stressful events in their recent past (6 months) were excluded from the study. Among the 62 subjects initially screened, 29 were eligible (age range: 57–79 y; *M*_age_: 68.13±1.28; *M*_BMI_: 25.86±0.57), and these participants were enrolled in the period 2012–2013 after signing an informed consent form (see Table 1 for the participants’ characteristics). The study was approved by the Ethis Committee of the University of Urbino. A schematic flowchart of the research participants is shown in Fig 1.

**Table 1:** Characteristics of the participants.

	High Fitness group	Low Fitness group	*p*-value*
**Age [years]**	64.57 (1.52)	70.26 (1.50)	0.01
**BMI [kg/m** ^**2**^ **]**	25.04 (0.80)	26.25 (0.33)	0.13
**VO**_**2max**_ **[mL/kg/min]**	37.92 (0.94)	26.38 (0.69)	0.00
**VO**_**2max**_ **reference percentile**	74.60 (2.38)	24.00 (1.75)	0.00

Characteristics of the participants belonging to the high-fitness (HF, *n* = 10) and low-fitness (LF, *n* = 12) groups. Student’s t-test analyses revealed significant between-group differences for mean age and minor, non-significant, differences for BMI. Mean values of estimated VO_2max_ and age-specific percentile of reference (retrieved from [36]) are also shown. Data are reported as mean values (±SEM).

* Student’s t-test.

### Cardiorespiratory fitness assessment and group assignment

The cardiorespiratory fitness assessment was used for group assignment purposes and was performed using the Rockport Walking Test. This submaximal test, originally introduced and validated by Kline and colleagues [37] and lately improved and adopted in several studies [38, 39], is largely supported by the literature (as reviewed by Noonan and Dean [40]) and is well suited for sedentary and/or older adults. Briefly, the participants were required to walk 1,600 m (jogging and running were not permitted) as quickly as possible while wearing a heart rate monitor (Polar, Kempele, Finland). The time needed to cover the distance was recorded, and the heart rate was measured immediately at the end of the test (subject standing). Walking time and heart rate were computed in an age-specific regression equation allowing for the estimation of VO_2max_. Estimated values of VO_2max_ were then confronted with age- and gender-specific percentiles of VO_2max_ distribution in the general population [36], and the participants were assigned to three groups: high-fit (HF, ≥60° percentile, *n* = 10), intermediate-fit (IF, 36°-59° percentile, *n* = 7), and low-fit (LF, ≤35° percentile, *n* = 12). Only the HF and LF subjects were selected to complete the study and proceed with the analysis of circadian and stimulated cortisol secretion (see below).

Selected participants were then asked to choose one activity category that described their usual pattern of exercise, and were classified as physically active when they met the following criteria: engaging in vigorous-intensity exercise at least 3 time per week for at least 20 minutes per session, or engaging in moderate-intensity exercise for 30 minutes at least 5 times per week for the past 3 years. Participants who did not meet the established guidelines for physical activity [41] were classified as physically inactive.

### Salivary cortisol detection and data analysis

The procedures were conducted as previously described [42]. At least 1 mL of saliva was collected in a Salivette® (Sarsted Aktiengesellschaft & Co., Nümbrecht, Germany). The subjects were instructed not to consume water or food (including candies or chewing gum), and to not brush their teeth within 30 min prior to sample collection. The saliva samples were centrifuged at 1000 rpm for 2 min, and the supernatant was collected and stored at -20°C. A commercial enzyme immunoassay kit to determine salivary cortisol (Diametra, Italy) was used according to manufacturer’s instructions. Cortisol concentration was expressed as nmol/L. The lower limit of detection for the assay was 0.5 nmol/L and the upper limit of the standard curve was 1750 nmol/L.

To investigate the circadian pattern of basal cortisol secretion, 6 salivary samples were collected. Since in most healthy people the morning awakening is associated with a brisk increase of cortisol secretion by about 40–80% of waking levels, reaching its peak around half hour after wake-up (cortisol awakening response, CAR; [43]), the first saliva samples were collected rigorously 30 min after awakening to ensure that the morning peak of cortisol secretion was not missed (although the lack of a wake-up measure hampered the analysis of the incremental phase of the CAR). The participants were instructed to start collecting samples only if: i) they had a normal duration and good quality of sleep the previous night; and ii) they woke-up between 6.30 and 8.00 h. The other samples were collected at 12.00, 15.00, 18.00, 21.00, and 24.00 h. The total daily cortisol output was indexed as the area under the curve with respect to ground (AUC), and was calculated with the trapezoid formula described by Pruessner and colleagues [44].

On the following day, between 2.00 and 3.00 PM, the cortisol response to mental stress was investigated. The subjects were first accommodated in quiet and relaxed conditions and told about the task procedure. The mental challenge had a duration of 5 min, and consisted of a color-word interference Stroop test (3 min), followed by a 2-min mental arithmetic task (counting backwards from 2083 in 17 steps as quickly as possible, and starting all over again in case of miscalculation). To increase the level of situational stress, the test was performed in front of an evaluative audience (2 men and 1 woman). It is worth noting that: i) a modified Stroop test administered to a mixed population of young and old women has been shown to trigger a small, but significant, increase in salivary cortisol, which was most pronounced in older women [45]; and ii) multiple-task stress protocols, including color-word Stroop and mental arithmetic tests, were able to trigger a significant increase in urinary [46] and plasma cortisol [47] in healthy women of different ages. Salivary samples were collected at 4 time points across the task: 10 min before starting the test (baseline, s1), 5 min after task completion (s2), and then after 30 (s3) and 60 min (s4). The acute cortisol response to mental stress was quantified by measuring: i) the baseline-to-peak increment of salivary cortisol concentration (nmol/L); ii) the area under the curve with respect to increase (AUC_I_), calculated as the difference in the area under and above the curve with reference to the baseline value [48]; iii) the slopes of cortisol rise (s1-to-peak) and recovery (peak-to-s4); iv) and the ‘peak time’ of the cortisol response, i.e. the time point at which a subject had the highest cortisol level, calculated by converting each subject's peak value into the actual minutes of the experiment (s1 = 0 min; s2 = 20 min; s3 = 50 min; s4 = 80 min). In accordance with previous literature [49–51], the subjects were subdivided into responders (R) and non-responders (NR) on the basis of the amplitude of their cortisol response to mental stress. In the present study, a baseline-to-peak cortisol rise of 1.5 nmol/L was considered to be the cut-off value to separate R from NR. Such a criterion was recently shown to serve as an easily applicable and relatively accurate proxy to detect cortisol pulses in experimental designs involving the induction of acute stress in laboratory settings [52]. In accordance with previous reports [50, 53], the NR subjects were excluded from further analyses.

### Statistical analyses

The Shapiro-Wilk test rejected normality of data distribution for raw cortisol values, and cortisol data were log-transformed. The raw data are reported so as to be physiologically meaningful, and are presented as mean ± standard error of the mean (SEM).

WSANOVA testing (sampling time as factor) was used to compare the circadian pattern of basal cortisol secretion in the HF and LF groups. The effect size was calculated by Cohen’s *d* statistic. Data were adjusted for age and BMI as covariates by analysis of covariance (ANCOVA). Student’s t-test analyses were conducted at each time point. Between-group differences in the AUC for total daily cortisol output were assessed by Student’s t-test and ANCOVA (adjusting for age and BMI as covariates).

Among the responders from both the HF and LF, between-group differences in the cortisol response indices were assessed by ANCOVA testing, adjusting for age, BMI, and baseline (s1) cortisol as covariates.

## Results

A total of 22 subjects falling in either the HF (*n* = 10; VO_2max_ 37.92±0.94 mL/kg/min) or LF group (*n* = 12; VO_2max_ 26.38±0.69 mL/kg/min) were enrolled in the study (Fig 1). All the HF subjects met the established criteria for being regarded as physically active. The participants belonging to the LF group did not meet such criteria, and were all classified as physically inactive. As shown in Table 1, t-testing revealed between-group differences for mean age (64.57±1.52 *vs*. 70.26±1.50 for HF and LF, respectively; *p* = .01) and minor, non-significant, differences for BMI (25.04±0.84 *vs*. 26.25±0.33 for HF and LF, respectively; *p* = .13). Both parameters, i.e. age and BMI, were considered as potential confounders in further covariance analyses.

### Circadian cortisol secretion

**Fig 2:**
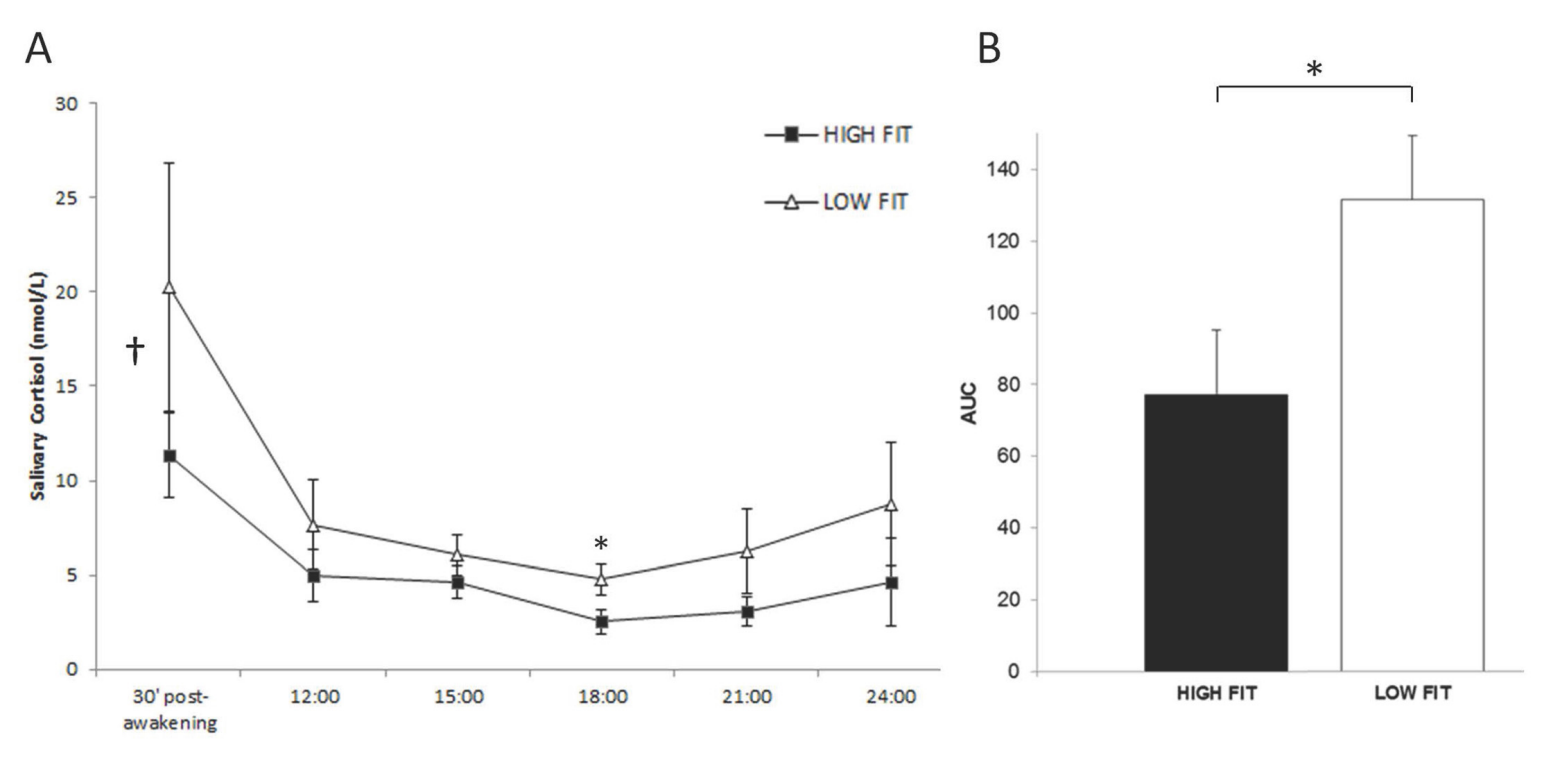
Cardiorespiratory fitness is negatively associated with basal cortisol secretion in healthy elderly men. (A) Salivary cortisol concentrations (nmol/L) measured at different sampling times during the day in high-fit (HF, *n* = 10) and low-fit (LF, *n* = 12) subjects. WSANOVA revealed a significant main group effect of aerobic fitness for cortisol levels overall [*F*(1,21) = 8.80, *p* < .05, Cohen’s *d* = -.54], as indicated by †. The effect was confirmed by ANCOVA testing, controlling for age and BMI as covariates [*F*(1,21) = 3.76, *p <* .05, Cohen’s *d* = -.54]. Significant group x time interaction (t-test; *p* < .05) is indicated by *. (B) The area under the curve values with respect to ground (AUC) for total cortisol output are lower in the HF participants (77.24±17.86 *vs*. 131.59±16.85 in HF and LF, respectively; t-test, *p* = .02). ANCOVA testing, adjusting for age and BMI, confirmed the effect [*F*(1,21) = 5.57, p < .05, Cohen’s *d* = -1.19]. In both (A) and (B), data are reported as mean values ± SEM. *, *p* < .05.

Salivary cortisol concentrations were found to be consistently lower in the HF than the LF subjects at all sampling times (Fig 2A). WSANOVA testing (sampling time as factor) confirmed a significant main effect of aerobic fitness for cortisol levels overall [*F*(1,21) = 8.80, *p* < .05, Cohen’s *d* = -.54]. ANCOVA testing, after controlling for age and BMI as covariates, further confirmed the effect [*F*(1,21) = 3.76, *p <* .05, Cohen’s *d* = -.54]. Student’s t-test analyses (Table 2) revealed HF-*vs*.-LF differences only at 18.00 h (*p* = .02), with differences at 30 min post-waking and at midnight being close to significance (*p* = .11 and *p* = .07, respectively). In addition, AUC values for total cortisol output across the day were significantly lower in the HF group (77.24±17.86 *vs*. 131.59±16.85 in HF and LF, respectively; Student’s t-test, *p* = .02; Fig 2B). ANCOVA testing, adjusting for age and BMI, confirmed this effect [*F*(1,21) = 5.57, *p* < .05, Cohen’s *d* = -1.19] (see individual, participant-level data points in the S1 File).

**Table 2:** Circadian pattern of salivary cortisol concentration.

Sampling time	High Fitness group	Low Fitness group	*p*-value*
**30 min post-awakening**	11.35 (2.26)	20.29 (6.59)	0.11
**12:00 h**	4.95 (1.36)	7.66 (2.38)	0.36
**15:00 h**	4.62 (0.88)	6.07 (1.07)	0.50
**18:00 h**	2.52 (0.62)	4.78 (0.81)	0.02
**21:00 h**	3.10 (0.78)	6.25 (2.25)	0.42
**24:00 h**	4.64 (2.34)	8.76 (3.25)	0.07

Salivary cortisol concentrations (nmol/L) measured at different sampling times in participants from the high-fit (HF, *n* = 10) and low-fit (LF, *n* = 12) groups. Between-group differences reach the threshold for significance at 18.00 h, and are close to significance at 30 min post-awakening and midnight. Data are expressed as mean (±SEM).

* Student's t-test.

### Cortisol response to acute mental challenge

A total of 13 subjects responded to the acute mental challenge (HF: *n* = 8, *M*_age_ 64.38±1.93, *M*_BMI_ 24.52±0.69; LF: *n* = 5, *M*_age_ 70.80±2.22, *M*_BMI_ 26.47±0.41). As reported in Table 3, mean values of the AUC_I_ and the baseline-to-peak cortisol increments were considerably higher in the HF group, with a steeper cortisol decline during recovery from mental stress, although the differences failed to reach the threshold for significance. The slope of cortisol rise and the mean peak time of cortisol response were very similar in the 2 groups (Table 3).

**Table 3:** Comparison if the cortisol response to mental stress in responders.

Measures	High Fitness group	Low Fitness group	*p*-value*
**AUC**_**I**_	120.38 (28.65)	76.99 (30.39)	0.12
**Cortisol increment (peak-s1) [nmol/L]**	6.83 (1.44)	5.40 (0.92)	0.08
**Mean peak time [min]**	38.75 (5.49)	38.00 (7.35)	0.55
**Rising slope (s1-to-peak)**	0.18 (0.03)	0.16 (0.02)	0.49
**Recovery slope (peak-to-s4)**	-0.30 (0.09)	-0.21 (0.63)	0.09

Comparison between the cortisol reactivity to mental stress in responders from the high-fit (HF, *n* = 8) and low-fit (LF, *n* = 5) groups. Between-group differences in the cortisol response indices were assessed by ANCOVA testing, adjusting for age, BMI, and pre-stress (baseline) cortisol levels as covariates. Data are reported as mean values (±SEM).

*ANCOVA testing, adjusted for age, BMI, and pre-stress (baseline) cortisol.

## Discussion

In summary, the results of the present study show that, in healthy elderly men, an active lifestyle and high cardiorespiratory fitness levels are associated with a lower diurnal cortisol output, with minor effects on the cortisol response to acute mental stress.

Previous evidence supports the view that HPA activity control worsens with aging. Among a cohort of nearly 3,000 community-dwelling individuals, subjects with an older age and lower physical functioning, notably males, have a higher risk of falling within the group showing a “raised curve” of salivary cortisol secretion, which is characterized by a higher cortisol total output and a flatter diurnal decline with respect to the “normative curve” [54]. Moreover, aging has been associated with increased daily cortisol AUC [6–8], a higher cortisol peak and nadir levels [8], an attenuated wake-evening slope and more pronounced cortisol awakening response [7], and an increased cortisol:DHEA ratio [6]. In the present study population, the total daily cortisol output was significantly lower in the high-fit than the low-fit subjects, after controlling for potential confounders such as age and BMI. This observation points to cardiorespiratory fitness as being a factor that is independently associated with better HPA control and lower basal cortisol levels, thus buffering the gradual HPA dysregulation that normally occurs with aging. Partly contrasting evidence comes from recent studies showing that lifetime exercisers and sedentary elderly men display similar cortisol concentrations in serum and saliva, despite remarkable between-group differences in VO_2max_ [28, 32]. In addition, it has recently been shown that habitual physical activity, although buffering the increase in the cortisol:DHEA ratio associated with stressful life events, does not affect salivary cortisol diurnal rhythms in older adults [29]. Differences in the study sample and methodology may explain such discrepancies. Hayes and colleagues [28, 32] used only a single sample of plasma or saliva in the morning to assess cortisol levels, thus giving no information on total daily output or diurnal rhythm of cortisol secretion. Meanwhile, in the work by Heaney et al. [29], nearly half of the participants suffered from a chronic illness, and the VO_2max_ was not determined. In addition, the patterns of physical activity reported by the subjects in the high-activity group showed high inter-individual variance, so that some participants might not have exercised at a sufficient level to had an independent impact on cortisol parameters.

In sedentary, elderly indiviudals, increases in VO_2max_ promoted by exercise programs are not necessarily associated with parallel modifications of cortisol secretion. After a 6-month aerobic training program, a reduction in serum cortisol levels was described in older women, but not in men [31]. Furthermore, a 6-week aerobic exercise program that was able to increase VO_2max_ in elderly men failed to affect salivary cortisol output [32]. A long-term perspective should thus probably be adopted to explain the negative association between VO_2max_ and cortisol production observed in the present study.

In this study, all high-fit subjects were physically active, i.e. they had regularly engaged in aerobic exercise over a long period of life. As a consequence, habitual physical activity may have relatively early and lasting effects on HPA across the lifespan, exerting long-term influences on cortisol regulation. In turn, long-standing levels of HPA activity and cortisol secretion may play a causal role in regulating VO_2max_. Cortisol is a catabolic hormone that stimulates degradation and inhibits the synthesis of muscle proteins, thus causing sarcopenia [55]. Chronically elevated levels of cortisol production may thus affect VO_2max_ due to the gradual loss of metabolically active muscle mass. In support of the latter view, alterations in circadian cortisol secretion have been found to be associate with several physical conditions that become manifest at an older age: higher cortisol levels have been associated with a worse physical performance in healthy older adults [56], and a positive correlation was found in elderly women between evening cortisol and increasing frailty [23]. In addition, longitudinal studies have proposed that lower night-time cortisol, together with greater diurnal drop in secretion, are associated with a better physical performance in later life [57, 58]. Interestingly, in the present investigation, we found that HF-*vs*.-LF differences in salivary cortisol reached the threshold of group x time significance in the evening samples, also remaining close to significance at midnight.

A growing body of evidence from cross-sectional [59–62] and prospective studies [18, 63–65] has shown that high levels of cortisol secretion correlate with poor cognitive outcomes in older adults (but see Singh-Manoux et al. [66] for an alternative view): men and women in the top quartile of baseline cortisol had a larger decline in cognitive functions measured over a 7-year follow-up period [18]; high diurnal free cortisol in serum has been associated with lower memory functions and processing speed over a 6-year period [64]; higher levels of cortisol output predicted poorer performance in executive functions and visual-spatial memory [65]. Negative correlation between cortisol levels and the thickness of prefrontal cortical areas has also been reported [12], providing neurobiological underpinning for the detrimental effect of excessive cortisol levels on cognitive outcomes. In light of these findings, present observation that habitual physical activity may play a role in contrasting the age-related dysregulation of HPA axis activity, acquires clinical relevance. In partial support for this view, evidence obtained in older adults with a mild cognitive deficit has shown that a 6-month aerobic training program that was able to increase cardiorespiratory fitness yielded beneficial effects on executive control processes, accompanied by a reduction in plasma cortisol levels (although such effects were only observed in women) [31].

There is no general agreement in the literature as to whether HPA responsiveness to psychosocial challenges varies with increasing age. Studies have either supported [33, 67–69] or refuted [70, 71] the notion that cortisol reactivity to acute psychological stress is greater in older individuals. Major gender differences have been reported: age-related increase in acute salivary cortisol response was found in men, but not in women [68]. In contrast, higher HPA axis reactivity and greater plasma cortisol levels in response to psychosocial challenges have been found in older women compared to men [68, 69]. In the present study, possible associations between aerobic fitness and the cortisol response to acute mental stress were evaluated in elderly men, with the results indicating that high-fit subjects tend to exhibit a larger response (indexed as baseline-to-peak increment and AUC_I_) and a steeper cortisol decline during recovery compared to their low-fit counterparts. Between-group differences, albeit showing a clear trend, failed to reach the threshold for significance. This was perhaps due to the small sample size and/or to the specific task we utilized to elicit stress, given that the Stroop test, compared to other mental tasks commonly used for inducing stress in humans (i.e. Trier Social Stress Test), is regarded as a weak activator of HPA axis [72].

These findings, pointing to a role, albeit minor, of cardiorespiratory fitness in affecting the cortisol response to acute mental stress in older men, are in contrast to previous evidence obtained from elderly women, which showed that high levels of cardiorespiratory fitness are associated with a lower cortisol response to psychological stressors [33]. Along with methodological differences in the experimental design and laboratory stressors, gender differences in the HPA axis responses to acute stress [68, 73, 74] may partially explain such discrepancies. Negative feedback in the HPA axis may become impaired with aging, thus contributing to age-related alterations of the stress response [13, 14]. Interestingly, we found that cardiorespiratory fitness seems to accelerate the recovery phase of the stress-evoked cortisol response, thus pointing to a more efficient feedback control on the acute HPA axis reactivity in high-fit individuals. In aging men, cardiorespiratory fitness may thus be associated with a more physiological and dynamic pattern of adrenal activation during acute mental stress.

### Limitations

Some limitations of the present study need to be pointed out. First, only males were enrolled, thus hampering any direct, between-gender comparisons and limiting the generalizability of the present results to the aging population overall. Second, individual values of VO_2max_ were estimated using a well validated submaximal walking test, and were not directly measured. However, since the two groups we confronted were very separate in terms of cardiorespiratory fitness levels, we believe that possible minor imprecisions in VO_2max_ estimation are unlikely to affect the interpretation of the results. Third, the present results on the basal cortisol output are based on repeated daily hormone assessments over 1 day, whereas measurements covering multiple-day periods are strongly advised in order to increase the reliability and generalizability of results [7]. In addition, a certain degree of participant non-adherence cannot be excluded, as the times when the salivary samples were collected were self-reported by the study participants, and the authors were unable to verify them directly. Fourth, we did not collect information on sleep-related variables, such as sleep duration and waking time, thus hampering their use as potential confounders in the statistical analyses. In fact, previous evidence has documented that cortisol levels and slopes may vary with waking time and the duration of sleep the previous night: CAR, cortisol peak values, and AUC have beeb reported to be higher in early awakeners, independent of age and gender [8, 75, 76]. Moreover, CAR and cortisol diurnal levels have been found to be elevated after a short sleep duration and increased sleep disturbance in a large community-dwelling population of middle-aged men and women [77]. However, it is worth noting that our participants were instructed to start collecting salivary samples only if: i) they had a normal duration and good quality of sleep the previous night; and ii) they woke-up between 6.30 and 8.00 h, i.e. within a rather restricted time interval that excluded extreme inter-individual variability in waking times. We are, therefore, inclined to believe that sleep-related variables are unlikely to introduce major bias to the interpretation of the present results. In addition, it was recently shown that the timing of cortisol peaks (and also nadirs) is not affected by non-extreme differences in waking time [8], thus reinforcing the assumption that the first saliva sample, collected at 30 min post-waking, reliably represents the peak of CAR in our study population.

## Conclusions

In summary, the results of the present study indicate that habitual physical activity and high cardiorespiratory fitness levels can positively affect HPA activity in healthy aging men, as the daily cortisol total output is significantly lower in high-fit than low-fit individuals. In addition, high-fit elderly men tend to exhibit a more dynamic pattern of cortisol responses to acute mental stress, thus pointing to better control of the HPA axis reactivity. Regular aerobic exercise may contribute to buffering the gradual HPA dysregulation that normally occurs with advancing age, thus favoring resilience to stress and contrasting age-related cognitive and physical decline in the aging population.

## Supporting Information

S1 File Participants' data. Individual, participant-level data points of circadian salivary cortisol concentration in high-fit (Table A) and low-fit (Table B) subjects.
(DOCX)
