# Supplementary material for: High Cardiorespiratory Fitness Is Negatively Associated with Daily Cortisol Output in Healthy Aging Men
Source: PLoS One. 2015 Nov 3;10(11):e0141970. doi: 10.1371/journal.pone.0141970 (PMC4631391; doi:10.1371/journal.pone.0141970)
Supplement: S1 File — Individual, participant-level data points of circadian salivary cortisol concentration in high-fit (Table A) and low-fit (Table B) subjects. (DOCX) [file pone.0141970.s001.docx]

**S1 File. Participants' data.** Individual, participant-level data points of circadian salivary cortisol concentration in high-fit (Table A) and low-fit (Table B) subjects.

**Table A**

|  | **BMI** | **AGE** | **CIRCADIAN SALIVARY CORTISOL (nmol/L) – High-Fit subjects** | | | | | |
| --- | --- | --- | --- | --- | --- | --- | --- | --- |
| **ID#** | **(kg/m^2^)** | **(years)** | **30’ post-awakening** | **12:00 h** | **15:00 h** | **18:00 h** | **21:00 h** | **24:00 h** |
| 220845 | 22.45 | 65.98 | 2.80 | 0.32 | 2.06 | 0.82 | 0.77 | 0.51 |
| 200738 | 26.22 | 73.07 | 23.52 | 13.35 | 6.28 | 2.54 | 2.81 | 25.42 |
| 230547 | 29.80 | 64.53 | 1.81 | 2.18 | 2.25 | 0.57 | 6.32 | 1.04 |
| 241149 | 24.68 | 62.02 | 25.54 | 9.97 | 8.83 | 5.60 | 5.46 | 3.14 |
| 180348 | 22.63 | 63.73 | 8.44 | 3.02 | 2.37 | 1.66 | 1.31 | 1.42 |
| 091048 | 24.93 | 63.16 | 3.62 | 3.14 | 7.79 | 1.92 | 1.38 | 1.76 |
| 300449 | 27.17 | 62.59 | 8.39 | 4.48 | 2.13 | 2.09 | 1.61 | 3.81 |
| 311039 | 22.91 | 72.09 | 7.51 | 2.37 | 6.91 | 1.88 | 1.93 | 3.24 |
| 140850 | 27.01 | 61.30 | 28.32 | 8.95 | 5.88 | 6.53 | 7.79 | 3.35 |
| 150954 | 22.61 | 57.21 | 3.57 | 1.68 | 1.75 | 1.58 | 1.66 | 2.68 |
| BMI, body mass index. | | | | | | | | |

**Table B**

|  | **BMI** | **AGE** | **CIRCADIAN SALIVARY CORTISOL (nmol/L) – Low-Fit subjects** | | | | | |
| --- | --- | --- | --- | --- | --- | --- | --- | --- |
| **ID#** | **(kg/m^2^)** | **(years)** | **30’ post-awakening** | **12:00 h** | **15:00 h** | **18:00 h** | **21:00 h** | **24:00 h** |
| 100245 | 25.71 | 65.66 | 19.61 | 14.66 | 6.70 | 9.13 | 8.11 | 5.27 |
| 250543 | 27.23 | 68.01 | 15.56 | 5.50 | 5.58 | 8.92 | 11.02 | 11.36 |
| 200546 | 26.18 | 65.45 | 88.76 | 5.55 | 0.94 | 1.70 | 0.75 | 5.17 |
| 280637 | 27.48 | 74.17 | 7.33 | 1.94 | 2.36 | 1.75 | 0.56 | 42.95 |
| 300846 | 26.68 | 65.04 | 18.10 | 4.90 | 7.14 | 2.66 | 1.80 | 6.64 |
| 290933 | 26.67 | 75.88 | 6.60 | 30.91 | 9.08 | 2.97 | 28.64 | 3.25 |
| 050646 | 27.78 | 65.20 | 11.04 | 3.18 | 2.16 | 2.26 | 0.83 | 6.11 |
| 050737 | 25.35 | 73.21 | 5.91 | 1.61 | 2.21 | 2.78 | 3.02 | 12.17 |
| 270732 | 24.82 | 74.05 | 25.67 | 9.64 | 8.74 | 8.04 | 5.33 | 5.86 |
| 210336 | 25.47 | 75.04 | 27.02 | 0.77 | 11.17 | 4.89 | 2.10 | 2.53 |
| 080544 | 27.40 | 67.11 | 12.84 | 6.13 | 4.77 | 5.74 | 6.44 | 2.56 |
| 270935 | 24.22 | 74.29 | 5.09 | 7.11 | 11.94 | 6.54 | 6.41 | 1.27 |
| BMI, body mass index. | | | | | | | | |
